# Assessing the Impacts of Preanalytical Field Sampling Challenges on the Reliability of Serum Aflatoxin B1-Lysine Measurements by Use of LC-MS/MS

**DOI:** 10.3390/toxins14090612

**Published:** 2022-09-01

**Authors:** Nicholas C. Zitomer, Michael E. Rybak, Maya R. Sternberg

**Affiliations:** Nutritional Biomarkers Branch, Division of Laboratory Sciences, National Center for Environmental Health, US Centers for Disease Control and Prevention, Atlanta, GA 30341, USA

**Keywords:** aflatoxin, aflatoxin B1-lys, AFB1-lys, mycotoxins, biomarker

## Abstract

Aflatoxin exposure is endemic in developing countries with warm, humid climates that promote toxigenic mold growth on crops and foodstuffs. Estimating human aflatoxin exposure is key to identifying and abating contamination sources. Serum aflatoxin B1 bound to albumin lysine (AFB1-lys) is a preferred exposure biomarker, but field sample collection, processing, transportation, and storage logistics are challenging. We validated an improved LC-MS/MS method for serum AFB1-lys and applied it to three field sampling challenges: transportation/storage (elevated temperature); collection/processing (hemolysis); and sample type substitution (heparinized plasma). Our new LC-MS/MS method had a LOD of 0.03 ng/mL, accuracy (mean spike recovery) of 112%, total imprecision (replicate pool measurements) ≤5% at ≥0.2 ng/mL, and results that were 95.1% similar (mean percentage similarity) to an established method. AFB1-lys in human serum spiked with serum from aflatoxin-dosed rats was stable for 14 days at both ambient (22.5 °C) and elevated (38 °C) temperatures. Simulated hemolysis (adding 0.25–3 mg hemoglobin) did not affect AFB1-lys accuracy at ≥0.5 ng/mL but caused 10–25% signal suppression. Heparinized plasma AFB1-lys was 99.0% similar to serum but interfered with albumin measurements (bromocresol green) causing spurious low bias. Further investigation is warranted, but our findings suggest that AFB1-lys is pre-analytically robust.

## 1. Introduction

Aflatoxins are mycotoxins produced by *Aspergillus spp*. that exhibit human hepatotoxicity, immunotoxicity, and teratogenicity. Many grains, nuts, and seeds are susceptible to infection and contamination during growth, harvest, processing, transport, and storage. Developing countries with warm, humid climates are particularly vulnerable to aflatoxin exposure where toxigenic *Aspergillus spp*. growth can thrive [1]. Estimating human aflatoxin exposure in these settings is key to improving public health by identifying and abating persistent sources of aflatoxin contamination. Aflatoxin B1 bound to the lysine residues of serum albumin (AFB1-lys) is a preferred biomarker of aflatoxin exposure. AFB1-lys has a circulating half-life of approximately 3 weeks post-exposure, offering a larger window for exposure detection in comparison to other biomarkers that are more rapidly excreted [2]. AFB1-lys has been quantified by the use of methods with measurement specificity ranging from low (ELISA, [3]), to intermediate (HPLC-fluorescence, [4,5]), and high (LC-MS/MS, [6]). LC-MS/MS is often preferred due to its superior measurement sensitivity and selectivity relative to other techniques [7]. 

The preanalytical phase of a laboratory test is the most vulnerable component of the entire testing process and is often performed outside of the immediate oversight and control of the testing laboratory [8]. Studies of aflatoxin exposure biomarkers are often conducted in developing countries where aflatoxin exposure is endemic [9,10]. Field sample collection, processing, storage, and transportation can be logistically challenging in these settings, and compromises in any of these steps may affect the reliability of the biomarker measurement obtained and confound the interpretation of the aflatoxin exposure being evaluated. In this report, we present the performance of an updated LC-MS/MS method for the determination of AFB1-lys in serum and apply it to study the effects of common yet understudied preanalytical challenges in aflatoxin exposure studies: hemolysis during sample collection, elevated storage temperatures during storage and transport, and substitution of heparinized plasma for serum samples.

## 2. Results

### 2.1. Method Development

**Figure 1:**
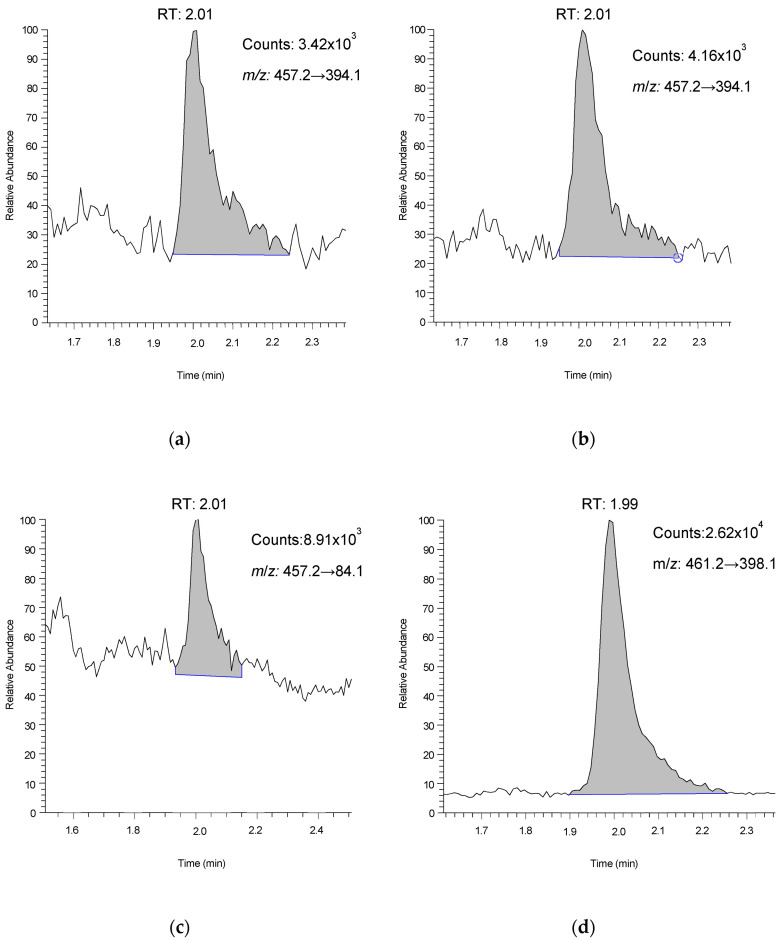
MS/MS chromatograms of AFB1-lys. (**a**) 0.05 ng/mL serum and (**b**) 0.05 ng/mL calibrator in phosphate-buffered saline, quantitation transition (*m*/*z* 457.2→394.1); (**c**) 0.5 ng/mL serum sample, confirmation transition (*m*/*z* 457.2→84.1); (**d**) ^2^D_4_-AFB1-lys, internal standard transition (*m*/*z* 461.2→398.1).

Infusion of AFB1-lys in positive ion mode yielded the same protonated [M+H]^+^ precursor ion (*m*/*z* 457.2) as our existing method [6] and MS/MS parameter optimization confirmed that the same product ion (*m*/*z* 394.1, presumed to be [M-NH_3_]^+^) was the best candidate for quantitation. Infusion of ^2^H_4_-AFB1-lys yielded the analogous precursor and product ion pair of *m*/*z* 461.2 and 398.1. We were able to optimize the *m*/*z* 84.1 product ion (presumed to be [C_5_H_10_N]^+^) for evaluation as a potential confirmation transition. Chromatographic resolution of AFB1-lys was also improved by the use of UHPLC conditions. Using a binary gradient of methanol and water with 0.01% formic acid throughout we were able to elute AFB1-lys at a retention time (RT) of approximately 2.0 min (Figure 1). Within-run variation of the analyte RT was typically <1% CV. Serum blanks showed no underlying interferences at the analyte RT in the quantitation, confirmation, and internal standard MS/MS transitions. Non-analyte peaks in the quantitation transition were occasionally observed in patient samples but remained resolved from the analyte peak by optimizing the mobile phase formic acid concentration to 0.01% and preparing mobile phases immediately before use. The intensity of the confirmation transition (*m*/*z* 457.2→84.1) was approximately 10% of that of the quantitation (*m*/*z* 457.2→394.1), limiting our use of the confirmation transition to verifying higher concentration samples on a case-dependent basis (Figure 1). Our calibration curves were linear (1/*x* weighting) with R^2^ values of ≥0.995, and phosphate-buffered saline (PBS) provided equivalent calibration to the matrix (serum)-based calibrators. 

### 2.2. Imprecision, Accuracy, and Sensitivity 

We determined method imprecision for the quantitation by calculating the within-run, between-run, and overall CV at four quality control (QC) pool levels (P1 through P4) measured in duplicate across 20 independent runs (Table 1). Based on the performance of QC pools P2–P4, the total imprecision of the method is expected to be <5% CV when measuring AFB1-lys concentrations >0.2 ng/mL, with within-run and between-run imprecision accounting for approximately equal proportions of the total CV. Imprecision increased as concentration decreased to 0.1 ng/mL, nearing 15% CV overall with between-run imprecision becoming a greater contributing factor. We assessed method accuracy and specificity by calculating the average recovery of AFB1-lys added to QC pools P3 and P4 in triplicate at concentrations near 0.5, 1, and 2× their respective endogenous AFB1-lys concentrations over two independent runs. The average recovery across all replicates and runs was 112% (SD 7.4%) with recovery being closer to quantitative (100%) in the lower concentration pool.

**Figure 2:**
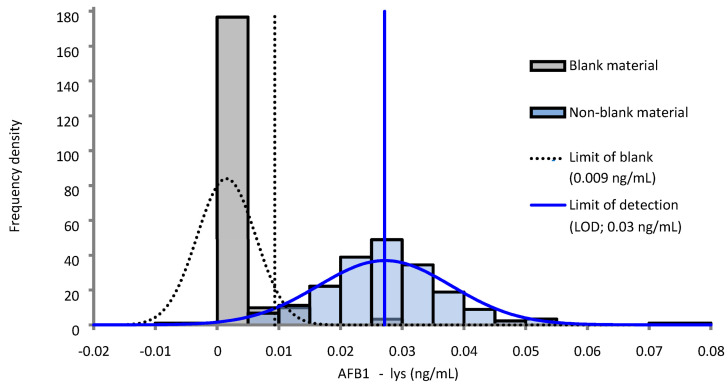
Probability distribution curve estimation of the limit of detection (LOD) [11] assuming false-positive and false-negative rates of 5% and data censoring at 0 ng/mL. Estimate obtained from 60 independent runs of a serum blank and two non-blank (~0.03 and 0.05 ng/mL) pools.

We calculated the method limit of detection (LOD) by a single repeat measurement of a serum blank and two low concentration (approximately 0.03 and 0.05 ng/mL) serum pools over 60 independent runs. The blank serum pool showed non-zero concentrations in 8 of its 60 measurements (13%) with only one of these instances yielding a concentration greater than the lowest calibrator concentration (0.030 ng/mL; lowest calibrator = 0.025 ng/mL). Assuming false-positive and false-negative rates of 5% and accounting for concentrations censored at 0 ng/mL by the instrument software we obtained a LOD of 0.03 ng/mL for the method with the limit of the blank estimated at 0.009 ng/mL (Figure 2) [11].

### 2.3. Method Comparison

**Figure 3:**
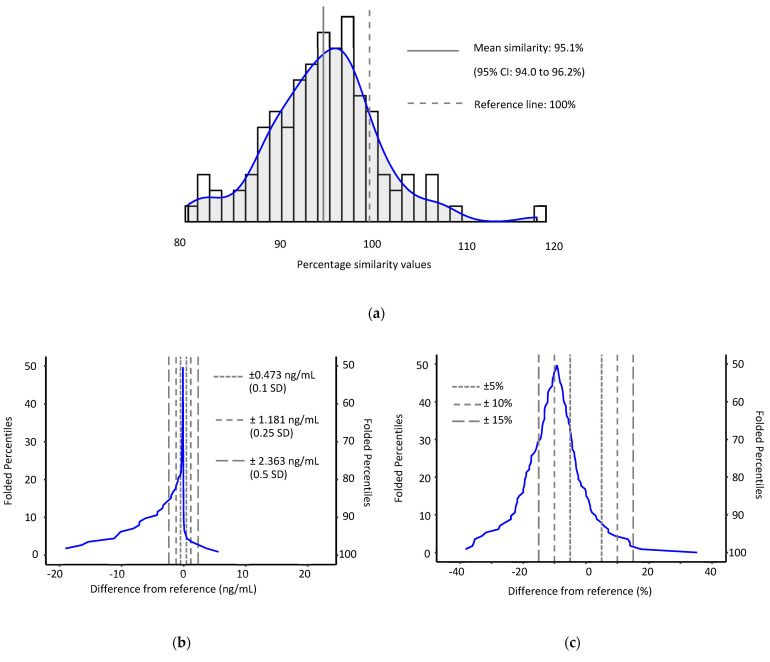
Comparison of new and established methods based on a convenience sample (*n* = 113). (**a**) percentage similarity histogram; (**b**) median concentration and (**c**) median % difference mountain plots.

Based on analyses obtained by the established method (*n* = 113), the concentration range for these samples was 0.06–176 ng/mL (median: 1.08 ng/mL). Sample concentration data from both methods were right skewed and log transformation did not adequately remove the skewness. Therefore, non-parametric methods were used for the purposes of method comparison. Results from both methods were highly correlated (Spearman *ρ* = 0.99, *P* < 0.001). A mean percentage similarity model, in which the average difference between our new and established methods is expressed as a percentage of the established method, showed that results from our new method were slightly lower, with a mean similarity of 95.1% (95% CI: 94.0 to 96.2%) to their counterparts. Calculating median differences between the two methods on both absolute and relative scales further confirmed a slight negative bias. The median concentration difference between methods was -0.086 ng/mL (95% CI: −0.114 to −0.064 ng/mL) with non-parametric 95% limits of agreement of −15.562 to 2.761 ng/mL. 83.1% of new method concentration results were within 0.5 SD of their established method counterparts. The median relative difference among methods was −9.2% (95% CI: −13.0 to −7.2%) with 95% limits of agreement of −35.4% to 13.97%. Moreover, 66.1% of new method concentration results were ±15% of their established method counterparts (Figure 3). Passing-Bablok regression yielded a similar relative bias of −7.5% (slope: 0.925; 95% CI: 0.879 to 0.961%) and an intercept of 0.018 ng/mL (95% CI: −0.046 to 0.014 ng/mL).

### 2.4. Effect of Prolonged Exposure to Elevated Temperature on AFB1-lys

The AFB1-lys concentration of serum samples appeared to be resistant to degradation under non-ideal storage conditions for up to two weeks. Our two-way ANOVA testing to determine the effect of exposing aliquots of QC pools to elevated temperatures (22.5 and 38.0 °C) and extended time periods (1, 2, 3, 5, 7, 10, and 14 days) did not reveal any statistically significant interactions over time. We saw a very small but statistically significant interaction by temperature with concentrations being marginally higher in serum stored at 38 °C versus 22.5 °C (Table 2). Owing to sample volume and resource limitations, we did not measure albumin in our 14-day temperature study of AFB1-lys-dosed QC pools; however, a set of three non-dosed human serum pools with no detectable AFB1-lys were subjected to the same time and temperature conditions. No statistically significant pool by temperature interactions were observed, and the pool by time interaction showed mixed results with two of the three pools showing significant (*P* < 0.0001) increases in serum albumin concentrations of up to 3.7% after 14 days (data not shown). 

### 2.5. Effect of Hemolysis on AFB1-lys

The presence of hemoglobin in serum samples appeared to have no effect on the accuracy of AFB1-lys concentration measurements; however, evidence of signal suppression was seen in the presence of hemolysis which may impact both sensitivity and imprecision. One-way ANOVA testing of added hemoglobin concentration (0.25, 0.5, 1, 2, and 3 mg hemoglobin/mL serum) on AFB1-lys concentration and internal standard peak area showed a significant overall effect (F-test *P*-value < 0.0001) for internal standard peak area in both pools (Table 3). Pairwise comparisons of the internal standard peak area for each of the hemolyzed conditions against the non-hemolyzed control found that the presence of hemoglobin at >0.25 mg/mL caused at least a 10% reduction in the internal standard peak area, with internal standard peak areas being reduced by 25% in worst cases (Table 4).

### 2.6. Effect of Substituting Heparinized Plasma for Serum

**Figure 4:**
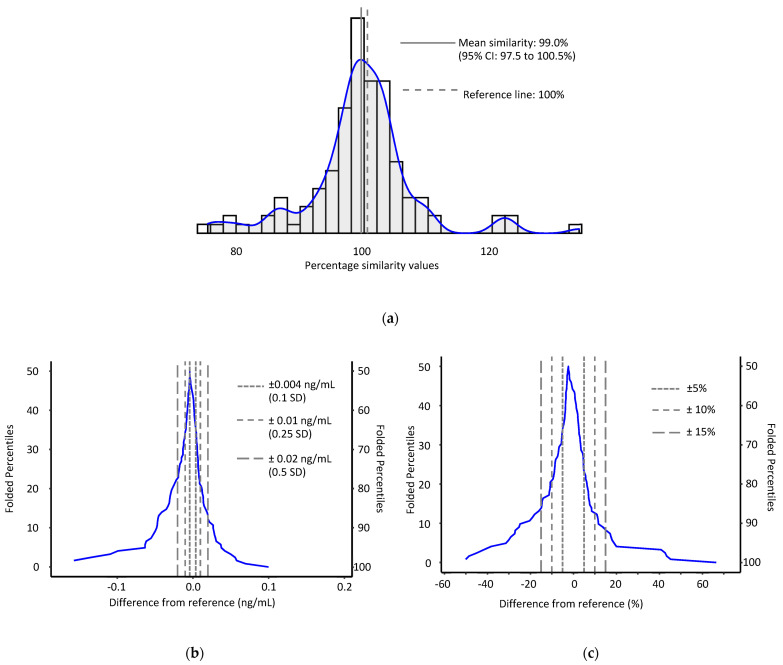
Comparison of AFB1-lys results from paired serum (reference) and plasma (test) samples (*n* = 122). (**a**) Percentage similarity histogram; (**b**) median concentration and (**c**) median % difference mountain plots.

We analyzed 225 pairs of serum and heparinized plasma samples drawn at the same venipuncture event. Approximately half (*n =* 122) had results that were ≥LOD and could be used for statistical comparison. Like our instrument comparison data, we found that both serum and plasma AFB1-lys concentrations in this convenience sample were right skewed and log transformation did not sufficiently correct this distribution; consequentially, we used non-parametric tests for comparison. AFB1-lys concentrations were highly correlated among the two sample types (Spearman *ρ* = 0.952, *P* < 0.001). Heparinized plasma generally yielded equivalent results for AFB1-lys when compared to serum. A mean percentage model of the data showed good agreement among the two sample types with plasma results being slightly lower than their serum counterparts, showing a mean similarity of 99.0% (95% CI: 97.5 to 100.5%) (Figure 4). Median differences between sample types similarly showed good agreement and slight negative bias. Plasma samples had a median AFB1-lys concentration difference of −0.004 ng/mL (95% CI: −0.007 to 0.002 ng/mL) with 95% limits of agreement of −0.109 to 0.056 ng/mL. Moreover, 65.6% of heparinized plasma concentration results were within 0.5 SDs of their corresponding serum results. The median relative difference in AFB1-lys for plasma vs. serum was −2.28% (95% CI: −3.67 to 1.30%) with 95% limits of agreement of −41.5 to 42.7%. Furthermore, 77.9% heparinized plasma results were within ±15% agreement of their serum counterparts (Figure 4). Passing-Bablok regression yielded a similar relative bias of −2.33% (slope: 0.977; 95% CI: 0.953 to 1.015) and a near zero intercept (1.26e^−4^ ng/mL; 95% CI: −5.32e^−3^ to 6.22e^−3^).

**Figure 5:**
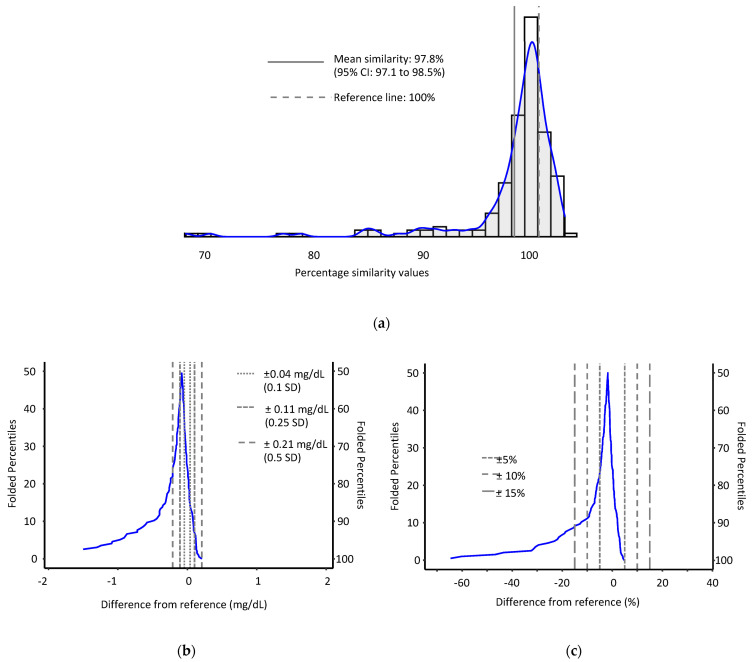
Comparison of albumin results from paired serum (reference) and plasma (test) samples (*n*=196). (**a**) Percentage similarity histogram; (**b**) median concentration and (**c**) median % difference mountain plots.

We analyzed the same 225 pairs of serum and heparinized plasma samples for albumin, of which approximately 87% of plasma samples (*n* = 196) yielded valid results free of interferences (100% of serum samples were reported). Heparinized plasma yielded albumin results that in most cases were lower than their serum counterparts. Compared to AFB1-lys, correlation in the albumin concentrations was much lower (Spearman *ρ* = 0.72, 95% CI 0.66–0.78, *P* < 0.001). Our mean percentage model showed for heparinized plasma similarity of 97.8% (95% CI: 97.1 to 98.5%) with evidence of low results for heparinized plasma (Figure 5). Plasma samples had a median albumin concentration difference of −0.07 mg/dL (95% CI: −0.10 to −0.06 mg/dL) with 75.5% of results within 0.5 SDs of their corresponding serum results, and a median relative difference of −1.78% (95% CI: −2.46 to −1.40%) with 77.9% of results being within ±15% agreement (Figure 5). The 95% limits of agreement for both median concentration differences (−1.325 to 0.131 mg/dL) and median percent differences (31.51 to 3.31%) suggested that the magnitude of the negative bias in some of the measurement pairs was substantial.

## 3. Discussion

### 3.1. LC-MS/MS Method Performance

In developing our updated LC-MS/MS method, we sought to leverage the improved chromatographic performance and MS detection sensitivity of an updated instrument to address the limitations and inefficiencies that were intrinsic to our existing method [6]. Our analyses, particularly in response to suspected aflatoxicosis outbreaks [12], are often performed under circumstances where serum sample volumes may be limited, sample quality may be non-ideal (e.g., hemolysis), repeat analyses for confirmation are needed, and the rapid and complete reporting of results is expected. In this sense, improving the sensitivity of our method was not a driving factor, and the LOD of our new method (0.03 ng/mL) is nominally equivalent to its predecessor [6] by design. This allowed us to conserve serum sample volume by reducing the assay serum volume requirement by 60% (150 μL vs. 250 μL), and greatly increase our ability to reinject samples by reducing the analysis injection volume by 90% (5 μL vs. 50 μL). Improved chromatography allowed us to reduce LC-MS/MS analysis times by 60% (6 min vs. 15 min between injections), permitting the analysis of 70 patient samples (100 injections including replicates of blanks, calibrators, and QCs) from 25 to 10 hours. Method imprecision was consistent with performance expectations, with total CV <5% at concentrations >0.2 ng/mL and <15% at concentrations approaching 3x the LOD (0.1 ng/mL). 

Results from both our new and existing methods compare well. Both methods had similar accuracy with spike recoveries in the same general range (100–115%) [6], and serum samples analyzed by both methods (*n* = 113) were highly correlated (Spearman *ρ* = 0.99, *P* < 0.001). Results from our new method were slightly lower with evidence of both absolute (−0.086 ng/mL) and proportional (−9.6%) biases. We hypothesize that this may be a product of chromatographic differences among the methods. By use of UHPLC conditions in our new method, we observed and resolved non-analyte peaks adjacent to the analyte signal in the quantitation MS/MS transition (*m*/*z* 457.2→394.1) that may not have been as well resolved in the previous traditional-HPLC separation [6]. 

### 3.2. Preanalytical Factors

The contamination of crops with fungal toxins such as aflatoxins is largely a product of challenges related to climate (i.e., elevated temperatures and humidity) and infrastructure (i.e., improper transportation and storage) [13,14], and these factors similarly pose challenges in terms of collecting, transporting, and storing biological samples collected to assess aflatoxin exposure in these settings. Our data suggest that AFB1-lys concentrations in serum samples are relatively robust and can withstand non-ideal transport and storage for up to two weeks at temperatures ranging from ambient room temperature (22.5 °C) to simulated elevated field temperatures (38 °C). We observed a small (≤0.03 ng/mL) but statistically significant increase in AFB1-lys concentration in two of the serum pools we studied. We hypothesize that this may have been due to small differences in evaporative loss between the temperatures studied exacerbated by our testing of relatively small sample volumes in tubes with non-threaded push-caps. An important qualification to the temporal stability we observed in serum AFB1-lys is that we obtained these data by use of human serum pools spiked with dosed rat serum. While highly similar in form, function, and stability, human and rat serum albumin have slight differences in molecular weight (66.4 vs. 65.9 kDa), amino acid sequence length (585 vs. 583 residues), and amino acid composition (73.0% identical), including the number of lysine residues (59 vs. 53) [15,16]. We believe it is unlikely that these differences in albumin composition would have a measurable effect on the observed stability of AFB1-lys, but future stability testing of human sera with endogenous AFB1-lys concentrations would be prudent to confirm AFB1-lys measurement stability. The mixed results we observed for serum albumin, with two of the three pools showing small (<4%) statistically significant concentration increases over two weeks, appears to be consistent with other studies. Studies in which serum samples were subjected to ambient room and transportation temperatures over timeframes of 4–5 days found that albumin concentrations were stable [17] or increased slightly (2.2%) [18]. It is important to state that the apparent stability of albumin-bound AFB1-lys in serum is not commutable to the storage of free AFB1-lys that would be used as a calibrator, and that stability can vary widely depending on the solvation conditions [19], nor should prolonged storage at room temperature or higher be substituted for the proven robust stability of low temperature (−80 °C) storage [20] when available.

Hemolysis is a leading cause of sample rejection in clinical laboratory analyses [21]. Inaccuracies arising from hemolysis have been documented in LC-MS metabolomics and proteomics, with non-targeted analyses being particularly susceptible [22], and a >0.5 mg/mL cutoff for sample rejection has been suggested [23]. The breakdown of red blood cells releases hemoglobin along with smaller quantities of other intracellular components that would not normally be present in a properly processed serum sample, and their spurious presence may interfere in LC-MS/MS measurement steps ranging from sample preparation (e.g., performance of proteolytic hydrolysis, SPE steps) to analysis (e.g., isobaric MS/MS interferences, signal suppression). To the best of our determination, the main effect that hemolysis (simulated in our experiments by the addition of hemoglobin) had on AFB1-lys measurement accuracy was signal suppression, and any impact on measurement accuracy was compensated for by normalizing the serum AFB1-lys quantitation peak area to the stable isotope-labeled internal standard. Despite this apparent correction for signal suppression, we caution against the analysis of hemolyzed serum samples without consideration of the AFB1-lys concentration being measured. Our experiments looked only at serum AFB1-lys concentrations >0.5 ng/mL; we did not measure samples at or near the LOD in our hemolysis experiments, and it is plausible that hemolyzed samples with concentrations at or near the LOD may erroneously appear as <LOD measurements because of the signal suppression encountered. In addition to the measurement of serum AFB1-lys, consideration also needs to be given to the effect of hemolysis on the serum albumin measurement. A study of serum hemolysis on routine clinical chemistry testing [24] showed that, while albumin results remained within a desirable bias of ±1.3% and differences were not statistically significant, increasingly negative measurement bias (0.5% to 1.4%) and additional CV (0.4% to 0.7%) may be encountered for hemoglobin concentration levels similar to what we tested (0.3 to 5.1 mg/mL). 

Serum is the preferred sample type for AFB1-lys analyses [7,9,10,24], as plasma samples may present interferences arising from the presence of fibrin and/or anticoagulant additives. The good correlation and agreement we observed among heparinized plasma and serum suggests that a heparinized plasma sample could be used for the AFB1-lys determination if serum was not available. We hypothesize that the proteolytic digestion in our AFB1-lys methodology likely addresses any potential interference from the presence of fibrin, and that the presence of the heparin additive itself does not interfere with proteolysis. However, the comparability of heparinized plasma albumin measurements with serum is also needed since an albumin concentration measurement is used by convention to normalize for the circulating albumin concentration from which the AFB1-lys analyte is proteolytically generated [25]. It has been documented that heparin can form insoluble precipitates with the bromocresol green reagent used in the colorimetric determination of albumin and can yield erroneously low results [26,27]. Our comparison of heparinized plasma and serum albumin measurements appeared to confirm this both by the lower degree of correlation, negative bias, and left-skewed distribution of differences in the albumin measurement comparison. Because of these observations, and the potential for generating spuriously higher AFB1-lys results when normalized to albumin, we do not recommend the use of heparinized albumin measurements determined colorimetrically by use of bromocresol green. The use of bromocresol purple [26] or additives with bromocresol green [27] has been shown to restore accuracy to heparinized plasma albumin measurements but we have not tested these approaches. The need for normalizing AFB1-lys measurements itself has been called into question [25], and so we believe further evaluation of heparinized plasma for AFB1-lys measurements is worth consideration.

## 4. Materials and Methods

### 4.1. Preanalytical Factors

HPLC or LC/MS grade solvents and 0.2 μm-filtered deionized (>18 MΩ∙c water (AquaSolutions, Jasper, GA, USA) were used to prepare all reagents and samples. Internal Review Board (IRB) approval was obtained for the collection and use of human serum and plasma samples and pools in our study, and AFB1-dosed rat serum was obtained following Animal Care and User Committee (ACUC) protocols.

### 4.2. Sample Preparation

Serum and plasma samples were prepared based on a previous method from our laboratory [6] modified to suit updated instrumentation. In brief, serum samples (150 µL) were aliquoted into a 96-well, 1 mL polypropylene collection plate (Waters Corporation, Milford, MA) and amended with 200 µL of 0.01 M phosphate-buffered saline (Sigma, St. Louis, MO, USA), 75 µL of a 50 ng/mL solution of internal standard (^2^H_4_-AFB1-lys, as described in [6]), and 150 µL of a 13 mg/mL solution of proteases isolated from *Streptomyces griseus* (Pronase™, EMD Millipore, Billerica, MA, USA). Plates were then mixed and incubated for a minimum of 4 hours at 38 °C to allow hydrolysis of the AFB1-lys adduct to take place. After hydrolysis, samples underwent solid phase extraction (SPE) by use of a mixed-mode polymeric sorbent (OASIS Max, 96-well, 30 mg sorbent, 30 µm particle, 80 Å pore, Waters Corporation, Harbor City, CA, USA). An automated, 96-channel negative pressure SPE system (Zephyr G3 SPE, PerkinElmer, Waltham, MA, USA) was used to perform the SPE steps. The SPE plate was first conditioned with 1 mL of methanol followed by 1 mL of water. Samples were then diluted by the addition of 0.3 mL of water, mixed, and loaded onto the SPE plate. Samples were then washed in sequence with 1.8 mL of water, 1.0 mL of a 70% methanol-in-water solution, and 0.8 mL of methanol. Finally, samples were eluted into a new 96-well collection plate by two sequential 0.35 mL additions of 2% formic acid in methanol. Eluates were then dried under nitrogen (Biotage, SPE 96 Dual Dry, Uppsala, Sweden) and reconstituted in 0.1 mL of solution equivalent to the LC-MS/MS starting mobile phase composition (28% methanol in water, 0.01% formic acid). 

### 4.3. LC-MS/MS Analysis

LC-MS/MS analyses were performed by use of a UHPLC system consisting of a binary pump, autosampler, and temperature-controlled column compartment (Vanquish Flex, Thermo Scientific, West Palm Beach, FL, USA) coupled to a tandem quadrupole mass spectrometer (TSQ Altis, Thermo Scientific). A reversed-phase C18 column (Syncronis C18, 50 mm × 2.1 mm, 1.7 µm particle size) (Fisher Scientific, Suwanee, GA, USA) with a 2.0 µm in-line filter (KrudKatcher Ultra, Phenomenex, Torrance, CA, USA) was used for chromatographic separation. LC-MS grade solvents and reagents were used to prepare mobile phases. A binary gradient separation (A: 90:10 water/methanol, 0.01% formic acid; B: methanol, 0.01% formic acid) was used to chromatographically resolve the AFB1-lys peak. The gradient was isocratic at 20% B from 0–2.5 min at 0.4 mL/min, linearly increased to 95% B from 2.5–3 min at 0.4 mL/min, and isocratic at 95% B from 3–4 min at 0.5 mL/min. Each run was equilibrated for 1 min at starting conditions before analysis. The autosampler was set to 10 °C, the column compartment was 50 °C, injection volume was 5 µL, and needle wash was 50:50 acetonitrile/isopropanol (120 uL). Analysis throughput was 5 min/sample. The instrument was operated in +ESI mode with MS/MS transitions (*m*/*z*) 457.2→394.1 for AFB1-lys quantitation, 457.2→84.1 for confirmation, and 461.2→398.1 for the internal standard. Collision energies, lens RF potentials, and other MS/MS parameters were optimized by infusing standards. MS/MS monitoring was switched to *m*/*z* 200.0→100.0 in negative mode ESI during each run from 3.5–4 min as a preventative measure to reduce the likelihood of quadrupole charging over time. Data processing and instrument control was performed by use of the instrument software (Xcalibur v.4.2 Thermo). AFB1-lys sample concentrations were determined interpolating the peak area ratio of the quantitation transition to internal standard transition against a 7-point calibration curve of AFB1-lys in PBS ranging from 0.025–10 ng/mL generated for each run (1/*x* weighting). Samples with concentrations above the highest calibrator (10 ng/mL) were diluted with PBS and reanalyzed.

### 4.4. Statistical and Other Methods

We used SAS v9.4 (SAS Institute, Cary, NC, USA), R (R Foundation for Statistical Computing), and Analyse-it (Analyse-it Method Validation v6.01.1, Analyse-it, Leeds, UK) to perform statistical analyses related to method validation, analytical performance, and preanalytical factor experiments. Unless indicated otherwise, we performed all AFB1-lys statistical calculations on concentration (ng/mL serum or plasma) results obtained by interpolation of the quantitation to internal standard MS/MS peak area ratio by use of our new procedure described in the section above or our existing procedure [6]. Serum and plasma albumin concentration measurements were made by use of a bromocresol green-binding colorimetric assay performed on a clinical analyzer (Cobas c501, Roche Diagnostics, Indianapolis, IN, USA) [28].

### 4.5. Method Validation and Analytical Performance

Imprecision was evaluated by determining AFB1-lys concentrations in four QC pools prepared by amending human serum with AFB1-lys from the serum of experimentally dosed rats (Institute of Environmental and Human Health, Texas Tech University; Bloomberg School of Public Health, Johns Hopkins University). The four serum pools were prepared with approximate target concentrations of 0.1, 0.2, 0.5, and 0.9 ng/mL AFB1-lys, respectively, and analyzed in duplicate over 20 independent runs to determine the mean concentration, as well as the within-run, between-run, and total standard deviation for each pool (SAS, Analyse-it). In addition to establishing method imprecision, means and standard deviations from the QC pools were used as part of a multirule QC program to determine run acceptability based on duplicate analysis of the QC pools in subsequent analysis runs (SAS) [29]. Accuracy was assessed by amending the two higher concentration serum pools in triplicate at AFB1-lys concentrations approximating 0.5, 1, and 2× the endogenous pool concentration and calculating the recovery of the spiked amount. LOD was determined by single measurements of a serum blank and two serum pools adjusted with AFB1-dosed rat serum to concentrations near the LOD (0.03 ng/mL and 0.05 ng/mL) over 60 independent runs. LOD was estimated from these data by generating probability distribution density curves for both the blank and non-blank materials, and then determining the detection threshold assuming false-positive and false-negative rates of 5% while accounting for values truncated at zero concentration by the LC-MS/MS instrument (Analyse-it) [11]. Method comparability was evaluated by reanalyzing a subset of serum samples (*n* = 113) that had been analyzed by use of our previous method [6] and yielded a valid >LOD result. A percentage similarity model [30] and non-parametric comparisons (Spearman correlation, median differences, Passing-Bablok regression; Analyse-it, R) were used to compare results.

### 4.6. Preanalytical Factors Experiments

Three experiments were conducted to simulate plausible preanalytical issues that may be encountered in field studies of aflatoxin exposure. A 14-day temperature study experiment was performed to simulate conditions where serum samples collected for AFB1-lys may be significantly delayed in transit. Triplicate aliquots (500 μL) of QC pools 2–4, and single aliquots of the 0.05 ng/mL LOD pool were stored in 1.5 mL microcentrifuge tubes at either 22.5 °C (ambient laboratory temperature) or 38 °C (incubator) for time periods of 1, 2, 3, 5, 7, 10, and 14 days after which they were returned to −70 °C storage. All samples for each temperature studied were then thawed and analyzed for AFB1-lys in the same analytical run along with pool aliquots that had not been exposed to elevated temperatures. Two-way analysis of variance (ANOVA; SAS) was used to evaluate the effect of temperature and time. A hemoglobin spiking experiment was performed to simulate situations where serum samples may have experienced hemolysis during sample collection or processing. Triplicate aliquots of the two highest concentration QC pools were amended with human hemoglobin (Sigma-Aldrich, St. Louis, MO, USA) to concentrations of 0.25, 0.5, 1, 2, and 3 mg hemoglobin/mL serum to simulate varying degrees of hemolysis. One-way ANOVA (SAS) was used to evaluate the effect of hemoglobin. A series of paired serum and plasma samples (*n* = 225), with each sample pair collected at the same venipuncture event, were analyzed to evaluate the comparability of the two sample matrices for situations where a serum sample may not be available (e.g., field collection limitations. A percentage similarity model and non-parametric comparisons (Spearman correlation, median differences; Analyse-it, R) were used to compare results.

## Figures and Tables

**Table 1:** Method imprecision.

QC Pool	Mean AFB1-lys, ng/mL	Imprecision, % CV *		
		Within-Run	Between-Run	Total
P1	0.103	8.1	11.6	14.2
P2	0.178	3.5	2.9	4.6
P3	0.524	2.0	1.9	2.8
P4	0.864	2.1	2.4	3.1

* Analysis over 20 independent runs, two replicates per run.

**Table 2:** Two-way ANOVA analysis of serum AFB1-lys by storage temperature and time.

Variable	QC Pool	F-Test *P*-Value for Overall Effect	Estimated Average Difference in Serum AFB1-lys Concentration (95% CI), ng/mL
Temperature *	LOD	0.02311	0.009 (0.0017–0.0163)
	P2	<0.0001	0.0137 (0.0086–0.0187)
	P3	<0.0001	0.0334 (0.0205–0.0463)
	P4	0.0605	0.0223 (−0.001–0.0457)
			**F-test *P*-value for linear trend**
Time *	LOD	0.8387	0.5179
	P2	0.3829	0.0908
	P3	0.1452	0.6149
	P4	0.3309	0.6047

* Temperature: 22.5 (reference) and 38.0 °C. Time: 0 (reference), 1, 2, 3, 5, 7, 10, and 14 days.

**Table 3:** One-way ANOVA analysis of serum AFB1-lys and internal standard (IS) peak area by hemoglobin concentration.

Variable	Outcome, Units	QC Pool	F-Test *P*-Value for Overall Effect
Hemoglobin *	IS area, counts	P3	<0.0001
		P4	<0.0001
	AFB1-lys, ng/mL	P3	0.5514
		P4	0.9202

* 0.25, 0.5, 1, 2, and 3 mg hemoglobin/mL serum.

**Table 4:** Pairwise comparison of AFB1-lys internal standard peak area under hemolyzed versus non-hemolyzed conditions.

QC Pool	Hemoglobin, mg/mL	Mean Change in Internal Standard Peak Area from Control, % (95% CI)	*P*-Value	Bonferroni-Adjusted *P*-Value *
P3	0.25	−15.1 (−20.1 to −10.2)	<0.0001	<0.0001
	0.5	−18.4 (−23.3 to −13.4)	<0.0001	<0.0001
	1.0	−16.9 (−21.9 to −12)	<0.0001	<0.0001
	2.0	−22.1 (−27.1 to −17.1)	<0.0001	<0.0001
	3.0	−22.9 (−27.8 to −17.9)	<0.0001	<0.0001
P4	0.25	−18.9 (−25.8 to −12)	<0.0001	0.0003
	0.5	−10.5 (−17.3 to −3.6)	0.0063	0.0313
	1.0	−25.0 (−31.9 to −18.1)	<0.0001	<0.0001
	2.0	−16.2 (−23.1 to −9.3)	0.0003	0.0012
	3.0	−24.9 (−31.8 to −18)	<0.0001	<0.0001

* 5 comparisons per pool.

## Data Availability

Data presented in this work is considered to be method development, improvement, and/or validation. It is not considered to be public health data and is not publicly available per CDC Policy #CDC-GA-2005-14 “Policy on Public Health Research and Nonresearch Data Management and Access”.
